# Radial trunk-centred reference frame in haptic perception

**DOI:** 10.1038/s41598-018-32002-3

**Published:** 2018-09-10

**Authors:** Lucile Dupin, Vincent Hayward, Mark Wexler

**Affiliations:** 1 Laboratoire Psychologie de la Perception, CNRS and Université Paris Descartes, 75006 Paris, France; 2 Institut des Systèmes Intelligents et de Robotique, Sorbonne Université, 75005 Paris, France; 3 Institute of Philosophy, School of Advanced Study, University of London, London, UK

## Abstract

The shape of objects is typically identified through active touch. The accrual of spatial information by the hand over time requires the continuous integration of tactile and movement information. Sensory inputs arising from one single sensory source gives rise to an infinite number of possible touched locations in space. This observation raises the question of the determination of a common reference frame that might be employed by humans to resolve spatial ambiguity. Here, we employ a paradigm where observers reconstruct the spatial attributes of a triangle from tactile inputs applied to a stationary hand correlated with the voluntary movements of the other hand. We varied the orientation of the hands with respect to one another and to the trunk, and tested three distinct hypotheses regarding a reference frame used for integration: a hand-centred, a trunk-centred or an allocentric reference frame. The results indicated strongly that the integration of movement information and tactile inputs was performed in a radial trunk-centred reference frame.

## Introduction

Spatial perception in touch is an intrinsically active process. The direct contact of a fingertip with an object provides spatial cues that are inherently ambiguous if the location and orientation of the finger in space is left unspecified. Even if the tactile inputs that are locally retrieved from an object can be quite informative about the whole object, for instance through local curvature^1^ or surface orientation^2^, this information is rarely sufficient to enable the perception of shape since the region of skin contact is typically much smaller than the object itself. It is therefore necessary to retrieve spatial information over time, using hand movements such as contour following^3,4^, where the sequence of locally acquired items of information can be organized in a structured manner.

Here we address the question of the reference frame in which haptic perception resulting from the integration of movement and sensory inputs may be represented. In previous studies, a number of reference frames were found to contribute to haptic spatial perception^5,6^. As in vision, what is perceived by touch does not always strictly correspond to physical spatial properties^7–9^ and some of these observed discrepancies provide an opportunity to study the representation of haptic space in humans.

A case in point is when the task is concerned with the perception of orientation in space. When a person, with the eyes closed, is asked to orient a bar to assume a parallel relationship with respect to another bar, systematic deviations from the notion of Euclidian parallelism were observed^4,10^. These deviations may be seen as resulting from a combination of egocentric—relative to an observer’s body—and allocentric—external or absolute—reference frames^5^. In these studies the orientation of the hand influenced the patterns of deviations^11,12^, suggesting that haptic space might be represented in a combination of allocentric and hand-centred reference frames^13^. Any deviation encoded in a hand-centred reference frame would imply that what should be perceived to be parallel would depend on the orientation of the hand. Studies of orientation perception based on the tactile oblique effect—oblique lines are perceived with lower accuracy than vertical or horizontal lines^14^—have also implicated the role of body-centred reference frames. An orientation perceived to be vertical or horizontal depended on the body posture and particularly on that of the head^6,15,16^. In localisation tasks, the pattern of deviations from the properties of Euclidian space was found to be represented in a combination of egocentric and allocentric reference frames^17^, and to be relative to head and body posture^18^. When the perceptual tasks concerned shapes or angles, egocentric reference frames^19^, arm/hand-centred and head-centred reference frames^20^ all contributed to spatial perception.

Cutaneous inputs, proprioceptive inputs, and movement information were all found to contribute to shape perception or the perception of angular relationships^20^. Movement^21,22^, motor^23^, or proprioceptive^24,25^ information were also found to contribute to length and distance perception, and so did arm-hand configurations^11,26^ and head positions^27^. Depending on the task, each of these inputs could be relevant or available, and could potentially explain the diversity of reference frames previously observed.

**Figure 1:**
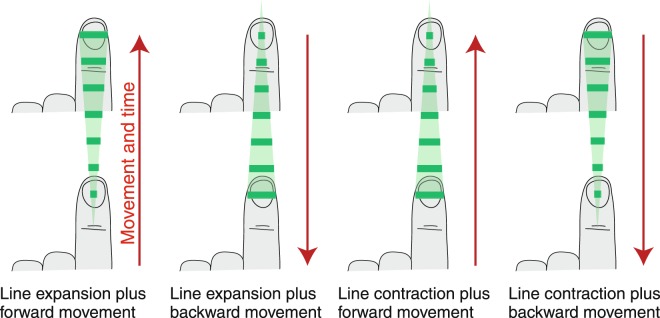
The four different combinations between movement (red arrow, forward or backward direction) and tactile stimulus (expanding or contracting line) when the movement and the sensory consequence originate from the same hand. The green triangle is the resulting perception of the coupling between each specific movement and tactile stimulus.

In the foregoing, we used a paradigm where tactile information was provided to one hand and movement information was provided by the other. Earlier, we discovered that the corresponding sources of spatial information were effortlessly integrated by participants to provide a unified perception of stationary shapes^22^. To be specific, if a person moved one hand along a straight path while a bar-like tactile stimulus oriented orthogonally to the movement was displayed to the contralateral fingertip and expanded or contracted proportionally to the hand movement, a stationary triangle was perceived. The natural combination of movement and tactile information within a single hand is represented in Fig. 1. If, however, the sources of information are assigned to different hands the combined perception of a triangle was the same as in the case of a single hand as if movement information was directly transferred to the stationary hand^22^.

**Figure 2:**
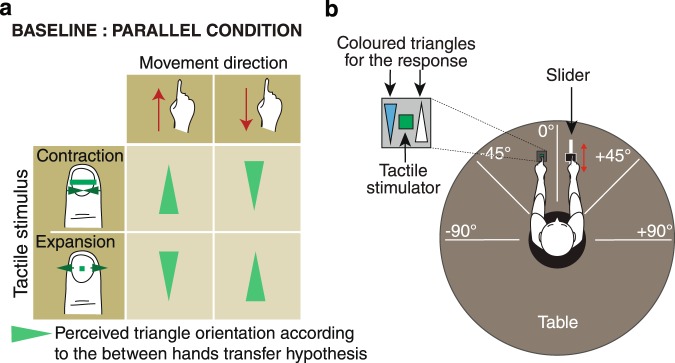
(**a**) Truth table of triangle orientations corresponds to the integration between one movement and one tactile stimulus in the baseline condition (between hands transfer). (**b**) Schematic view of the set-up on the experiment. The participant is located at the centre of a circular table. His/her right hand is positioned on the slider and his/her left hand on the tactile display. The two triangles blue or white are used for the participant to indicate perceived triangle orientation. Arm and hand positions represented here correspond to the baseline condition.

An important feature of this paradigm was that the perceived triangle orientation was a logic function of each of the binary inputs, as is illustrated in Fig. 2a, and was completely unpredictable. Thus, we found that movement information arising from one hand was combined with the tactile information experienced by the other hand as if the stationary hand was moving like the active hand. The tactile stimulus did not contain any information about the spatial extent and orientation of the triangle. The perceived orientation of the triangle resulted from the expansions or contractions of the tactile bar combined with the movements of the hand. In a subsequent study we showed that this type of information transfer generalised to different limbs^28^.

In these previous studies, hands were positioned in front of the participants so it was not possible to identify the reference frame in which the association between movement and tactile information took place. Integration in egocentric or allocentric reference frames would lead to the same result. We used the information combination paradigm in the present study with a set-up illustrated by Fig. 2b where a tactile stimulator generating expanding and contracting stimuli and a slider that constrained the hand movements of participants could be positioned to assume different relationships with respect to the trunk of participants. We could systematically vary the relative position of the hands with respect to one another and their positions with respect to the body. Through testing participants under different conditions, we tested three distinct hypotheses regarding the possible use of frames of reference; namely, the use of hand-centred, trunk-centred, or allocentric frames of reference.

**Figure 3:**
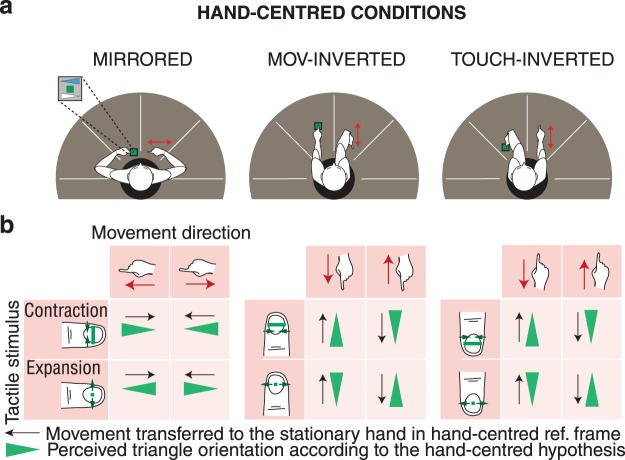
(**a**) Representation of the positions of arms and hands for the three conditions testing the hand-centred reference frame hypothesis. (**b**) For each condition above, the table of triangle orientation perception corresponding to a hand-centred reference frame.

The first hypothesis tested was that of a hand-centred reference frame for integration. According to this hypothesis, the participants would integrate the two inputs, each coded relative to the hand from which it originated, independently from the positions of the hands in space. A hand-centred hypothesis would predict that the perceived orientation of the triangle in absolute space would depend on the orientation of the hands in absolute space. To test this hypothesis, the orientation of one hand was inverted relative to the other and the two hands were oriented in space in three different manners, as it is illustrated by Fig. 3a. A movement of the right hand along the direction of the index finger would then be integrated with the tactile stimulus applied to the left hand as if the left hand was moving in the direction of the right hand’s index finger. Thus, the direction of hand movement would have to be inverted before integration with the tactile stimulus as indicated by Fig. 3b.

**Figure 4:**
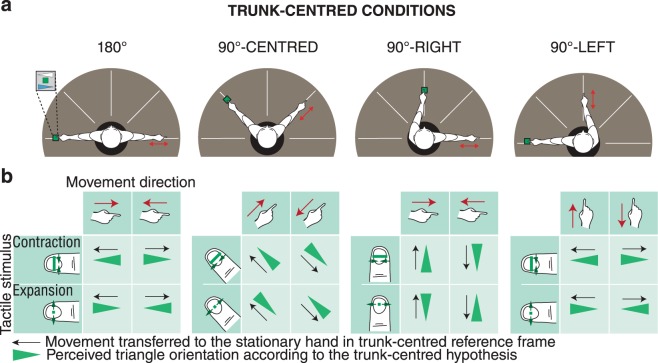
(**a**) Representation of the positions of arms around the body for the three conditions testing the trunk-centred reference frame hypothesis against the allocentric reference frame. (**b**) For each condition above, the table of triangle orientation perception corresponding to a hand-centred reference frame. In an allocentric reference frame hypothesis, triangle orientation will be perceived as opposite in 180° condition and at chance level in the three last conditions.

If the hypothesis of hand-centred reference frames could be rejected, then the two other possible reference frames for integration were trunk-centred or allocentric reference frames. To differentiate between these possibilities, we positioned the participants’ hands and arms in four different radial direction combinations as shown by Fig. 4a. If spatial information was integrated in a radial, trunk-centred reference frame, then an outward movement of the right hand would always be integrated as if the hand receiving the tactile information was moving outward. In other words, the results would be invariant under orientation changes around the trunk. The corresponding predictions are indicated by Fig. 4b. If, on the other hand, the reference frame of integration was allocentric, when the hands were oriented at 180° from one another, an outward movement of the right hand would be integrated with the tactile stimulation available at the left hand’s fingertip as an inward movement. The perceived orientation of the triangle would be the opposite from the case of a trunk-centred reference frame. In other conditions where hands were positioned at 90° from one other, the perception should be at chance level.

## Results

Between-participant standard deviations are indicated in brackets following the reporting of the mean.

### Baseline: Parallel condition

**Figure 5:**
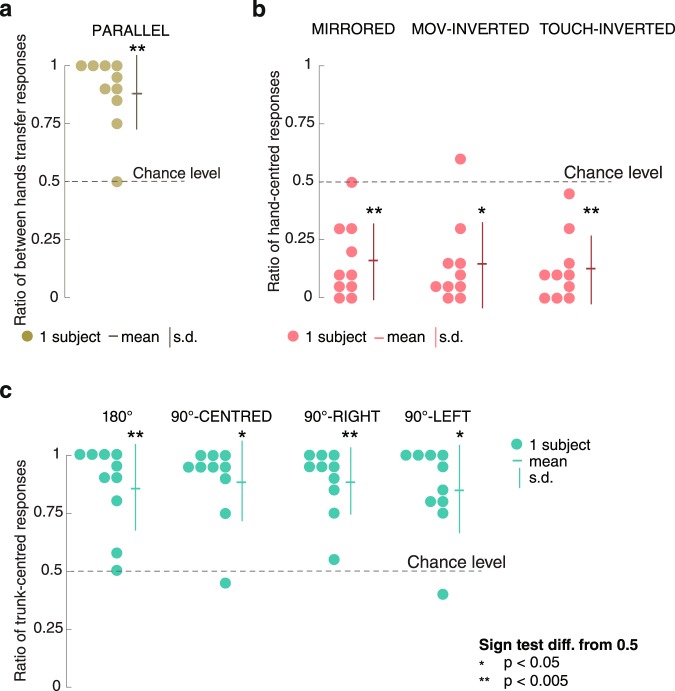
(**a**) Mean ratio of responses for each subject in the baseline condition corresponding to the between hands transfer hypothesis (Fig. 2a). (**b**) Mean ratio of responses for each subject in the three conditions corresponding to a hand-centred reference frame (Fig. 3b). (**c**) Mean ratio of responses for each subject in the four conditions corresponding to a radial trunk-centred reference frame (Fig. 4b).

For each participant, we calculated the ratio of responses in which motion direction was transferred from the right to the left hand, following the truth table of Fig. 2a. A between hands transfer response was coded by a 1 and the opposite response was coded by a 0. Results are collected in Fig. 5a. The mean over subjects was 0.89 [0.16]. The median score over subjects was 0.93 and was significantly different from the 0.5 chance level (two-tailed sign test: *p* = 0.004). This result replicated our previous findings^22,28^.

### Hand-centred reference frame hypothesis

For each trial, responses compatible with a hand-centred hypothesis (see Fig. 3b) were coded by a 1 and the opposite response was coded by a 0. Mean ratios for each subject and each condition are shown in Fig. 5b. The mean of responses over subjects corresponding to the hand-centred hypothesis was 0.16 [0.16] for MIRRORED, 0.14 [0.18] for TOUCH-INVERTED and 0.12 [0.16] for MOV-INVERTED. Median of responses over subjects was significantly different from the 0.5 chance level for all conditions using a two-tailed sign test (MIRORRED: median 0.1, *p* = 0.002, TOUCH-INVERTED: median 0.07, *p* = 0.02, MOV-INVERTED: median 0.12, *p* < 0.001). There were no significant differences between the three conditions (paired sign test, p ≥ 0.43 for all comparisons). The results for each condition excluded the hypothesis of a hand-centred reference frame.

### Trunk-centred reference frame hypothesis

Responses compatible with the trunk-centred hypothesis (Fig. 4b) were coded by a 1 and the opposite response was coded by 0. Responses according to allocentric reference frame hypothesis were coded by a 0 for 180° condition and were at chance level for all perpendicular arms conditions. Mean ratios for each subject and for all four conditions are presented in Fig. 5c. The mean of responses over subjects was 0.86[0.18] for 180°, 0.89[0.17] for 90°-CENTRED, 0.89[0.14] for 90°-RIGHT and 0.89[0.19] for 90°-LEFT. For all conditions the median score over participants was significantly different from the 0.5 chance level using a two-tailed sign test (180°: median 0.93 *p* = 0.002, 90°-CENTRED: median 0.95 *p* = 0.02, 90°-RIGHT: median 0.95, *p* < 0.001, 90°-LEFT: median 0.90 *p* = 0.02).

Using paired comparison, median scores for all conditions were not significantly different (paired sign test, p ≥ 0.69 for all comparisons). Results in all four conditions were compatible with the radial trunk-centred hypothesis and exclude the allocentric hypothesis.

## Discussion

While some studies, like ours, found evidence for the preferential role of trunk-centred reference frames in haptic spatial perception^6,29,30^, some other studies have argued for the contribution of hand-centred reference frames^11,12,20^. In parallelism matching tasks, where a role for hand centred-reference frames was found, the tactile information from the contact between the hand and the bars was not essential. This observation also applies to studies about the perception of hand orientations^31,32^. A misperception of hands orientation relative to the arm was observed when participants had to match orientation using a rod^32^. These discrepancies may be explained by the fact that these other studies employed different perceptual and motor tasks. For example, in geometric matching tasks, the movement and the position of the arms seemed to play a role in the misperception of orientations^26^. In the 180° condition shown in Fig. 4a, the position was similar to one condition of a previous parallel matching study^33^ in which the authors found that the deviation from parallelism of about 50°, less than half of the physical angle between hands. In the present set of experiments, a similar amount of hand-centred deviation would have no impact on performance. We cannot, however, exclude a minor contribution from hand-centred or allocentric reference frames. Another difference from previous studies is the absence of differences between symmetric and asymmetric positions around the body mid-sagittal plan, suggesting that the underlying integration mechanisms could be different.

In the current study, the spatial information originated from movement, but not from tactile information. The radial trunk-centred reference frame hypothesis, that we found to be well supported, could be linked to the reference frame of movement itself. In a study concerned with a similar question^34^, participants indicated line orientations simultaneously with the two hands. The pattern of interference suggested that hand movements were also encoded in trunk-centred reference frames and that there was no effect of asymmetry around the mid-sagittal plane, as in the present study. Evidence for the role of trunk- or body-centred reference frames was also found for leg movements^35^.

An egocentric reference frame based integration between movement and touch correlates strongly with recently identified functions of the posterior parietal cortex (PPC) in neuro-functional studies^36^. The PPC was found to contribute to spatial sensory-motor transformations. This role is particularly prominent in the right PPC, thought to support association between movement and tactile inputs both in the spatial remapping of touch^37^ and in the egocentric representation of space^36,38,39^. Finally, lesions in the PPC were observed to disrupt spatiotemporal exploratory movements and impair their integration with tactile inputs, leading to impoverished tactile object recognition^40,41^.

Taken together, our and previous results indicate that the spatial representation from touch clearly depends on movements. The sensorimotor integration that occurs in a trunk-centred reference frame could be useful for designing haptic interfaces when tactile feedback is not collocated with movement over the body.

## Method

### Participants

Ten volunteers participated in this experiment (mean age 23 [4.2], 4 males and 6 females, right-handed). All participants were naïve concerning hypotheses of the study and they were compensated 10€ for their time. All participants gave their written informed consent prior the experiment. The study was conducted according to the guidelines of the Declaration of Helsinki concerning research involving human subjects and was approved by the “Comité de protection des personnes Ile-de-France II” permit 2011-06-16.

### Apparatus

The tactile stimulations was generated using a tactile display: Latero Tactile Display from Tactile Labs^42^. The display is sized to stimulate the tip of one finger (area of 1.2 cm²). It consists of a matrix of 8 × 8 pins. Each pin can move laterally in one direction and independently from the others. The maximum amplitude of pin movements is 0.1 mm and bandwidth of about 100 Hz.

The linear slider used to record and monitor the movement of subjects was 23 cm long. An optical reader mounted on the slider allowed to retrieve with a negligible latency the position on the slider with a precision of better than 0.1 mm.

### Stimulus

The tactile stimulus consisted in a line on the fingertip, perpendicular to the finger, which could expand or contract depending of the trial (see Fig. 1 for a discrete view of the expanding or contracting line). The level of contraction and expansion depended on the position of the moving hand on the slider. The centre of the triangle was associated with the central position of the slider with a random jitter from 0 cm to +−1 cm. The distance of the movement on the slider between the beginning and the end of the tactile stimulation was the size of the triangle. There were two sizes: 8 and 16 cm (each size was associated with half of the trials for each block in randomized order).

### Task

Depending on the condition, participants sat on a stool or stood up in the centre of a circular table of diameter 210 cm with a circular hole of diameter 62 cm in its centre. The participants’ arms were positioned according to the condition to be tested. The head and trunk of the participants always faced the 0° angle of the table (Fig. 2b). The left index finger of the participants was positioned on the tactile display and their right index finger on the slider.

During each trial, with the eyes closed, participants performed one movement in a single direction using the right index finger to push a platform from one end of the slider to the other. The direction was alternated from one trial to the other. During the movements, a tactile stimulus, randomly contracting or an expanding line was presented to the left index fingertip. On each side of the tactile display two triangles were shown (see Fig. 2b): a white triangle pointing in the direction of the index finger and a blue one pointing in the opposite direction. The apex of the white triangle was always oriented in the direction indicated by the pointed index finger (see Figs 2b, 3a and 4a). To alert the participants the completion of the movement, a beep sounded when the end of the slider was reached. The participant then responded ‘blue’ or ‘white’ as a function of the perceived orientation of the triangle. The experimenter pressed the corresponding key on the keyboard of the experimental computer and validated the response prompting the participant to proceed with the next trial.

### Conditions

There were eight conditions, characterized by different arm/hand positions. Each condition comprised a block of twenty trials. Each trial combined a hand movement in alternating direction relative to the pointing direction of the index finger with an expanding or contracting tactile line stimulus. The eight conditions were divided in three groups, one baseline group and two test groups, each group corresponding to the testing of one hypothesis. The whole experiment started with the PARALLEL baseline condition. The seven remaining conditions were randomized.

#### Baseline

The PARALLEL condition was the baseline condition and a replication of previous studies. The participant sat on the stool. The arms and hands of the participants were positioned to be parallel, the two index fingers pointed away from the participant’s trunk (see Fig. 2b). The distance between the two hands was 30 cm. This condition tested the between hands transfer of tactile and movement information. The responses corresponding to this hypothesis are shown in Fig. 2a.

#### Hand-centred reference frame hypothesis

The hand-centred hypothesis was tested in three conditions, as illustrated by Fig. 3a. In all these conditions, the participants stood in the centre of the table to be able to position their arms as required. In the MIRRORED condition, the participants positioned their hand such that the index fingers pointed toward each other. The TOUCH-INVERTED and MOV-INVERTED conditions were like the PARALLEL condition but with lateral adduction of the wrist and of the arm 180° from the orientation of the hand resting on the tactile stimulator in the TOUCH-INVERTED condition and from the hand on the slider in the MOV-INVERTED condition.

#### Trunk-centred reference frame hypothesis

This hypothesis was tested using the conditions shown in Fig. 4a. The participants sat on a stool centred in the table. They aligned their hands with the axes of their forearms. The left index finger and the midpoint of the slider were positioned at 57 cm from the centre of the table. The orientation of each hand was radial with respect to the trunk. Angles for arm positions were indicated on the table’s surface. In the 180° condition, the participants positioned their right arm on the right side of their bodies at and +90° angle and their left hand at −90° angle (see Fig. 2b), giving an angle of 180° between the two hands. The 90°-CENTRED condition was like the 180° condition with the difference that the hands were positioned at −45° and 45° angles creating an angle of 90° between the hands symmetrically with respect to the sagittal plan. The two conditions 90°-LEFT and 90°-RIGHT were like the 90°-CENTRED condition but with a rotation of the two arms of −90° and +90° respectively around the trunk.

### Pre-test

The experiment was preceded by a test to ensure that the participants could experience the tactile stimuli. In this pre-test, the tactile display was positioned on the slider. With their right index finger, the participants moved the tactile display along the slider. There were twenty trials with the same settings as in the main experiment. The participants had to reach 80% correct responses over twenty trials for the triangle orientation before starting the experiment (see Fig. 1, for the correct responses). Seven participants succeeded at the first attempt, two at the second and one at the third.

### Statistical analyses

**I**ndividual mean perceptual judgments were compared to chance level (0.5) using the sign test to account for the non-normality of the data. Non-normality was assessed using the Kolmogorov-Smirnov test. The comparisons between conditions were analysed using the paired sign test with Bonferroni correction for multiple comparisons. All statistics were computed using Wolfram’s Mathematica 10.

## Data Availability

The datasets analysed during the current study are available from the corresponding author on request.
